# The Role of Place of Birth and Residence in Puerto Rican Health Disparities: Evidence From Disability Prevalence Among Archipelago- Vs. Mainland-Born Puerto Ricans

**DOI:** 10.1177/08982643231172643

**Published:** 2023-04-28

**Authors:** Mara Getz Sheftel, Frank W. Heiland

**Affiliations:** 1 Population Research Institute, 8082 Pennsylvania State University, University Park¸ PA, USA; 2 5920 Baruch College, New York, NY, USA

**Keywords:** disability, health disparities, social determinants of health, Puerto Rico Community Survey (PRCS), American Community Survey (ACS)

## Abstract

**Objectives:** This paper provides new estimates of disability prevalence for the archipelago and mainland-residing Puerto Rican populations ages 40 and above and compares disability by place of birth and place of residence to investigate drivers of middle and older age health. **Methods:** Large nationally representative samples from 2013 to 2017 American Community Survey and Puerto Rico Community Survey data are used to estimate age-specific disability prevalence for archipelago-born/archipelago-residing, archipelago-born/mainland-residing, mainland-born/mainland-residing Puerto Ricans. **Results:** Mainland-born/mainland-residing Puerto Ricans have the lowest age-adjusted disability rates and archipelago-born/archipelago-residing Puerto Ricans have the highest rates. Differences in education explain part of this disparity. **Discussion:** Similarities in disability prevalence are strongest based on where one was born as opposed to current residence, pointing to early life as a critical period in the disablement process for later-life health. Early life socio-economic disadvantage on the archipelago may have an enduring impact on later-life disability prevalence for archipelago-born Puerto Ricans.

## Introduction

Research on health outcomes of older age Latinos in the United States consistently shows that Puerto Ricans on the mainland have among the highest disability rates (Coustasse et al., 2009; Markides et al., 2007; Melvin et al., 2014; Sheftel, 2017). However, limited research looks at disability prevalence among older archipelago-residing Puerto Ricans (exceptions include: Payne, 2018; Pérez & Ailshire, 2017; Prina et al., 2020). This is despite the fact that the Puerto Rican population on the archipelago is experiencing rapid aging (Matos-Moreno, Santos-Lozada, et al., 2022) caused by decreasing fertility rates and morality rates, and a sudden increase in the outmigration of the working-age population (Palloni & Souza, 2013). The combination of these population dynamics may render older adults vulnerable to a protracted period of disability at older ages with limited caregiving resources. Moreover, there is a lack of research comparing older age disability prevalence between Puerto Ricans by place of birth and place of residence, well-known determinants of health for this population (Arévalo et al., 2015; Lee-Bravatti et al., 2021; Mendes de Leon et al., 2011). A comparison of disability rates within the older Puerto Rican population can inform our understanding of the social processes and exposures contributing to older Puerto Rican adult health and shed light on how they may manifest themselves in other populations.

The aims of this paper are two-fold. First, using two of the only large-scale, nationally representative datasets that can be combined to include the mainland- and archipelago-based populations of Puerto Ricans, we contribute new estimates of middle and older age Puerto Rican disability prevalence for those residing on the mainland and on the archipelago. Puerto Ricans comprise almost 10% of the Latino population age 40 and older living in the 50 US states and the Commonwealth of Puerto Rico.^1^ The distinctive sociopolitical history and colonial status of Puerto Rico have resulted in a lack of investment in public health and the outmigration of health professionals (Benach et al., 2019; Mulligan, 2014; Ramos et al., 2022). The history and political status of Puerto Rico may have implications for aging and disability trajectories. Updated estimates are critical to understand the current disability status of this aging population and anticipate future patterns and needs. Second, we compare disability prevalence of three groups of Puerto Ricans—mainland-born Puerto Ricans residing in the mainland, archipelago-born Puerto Ricans residing on the archipelago, and archipelago-born Puerto Ricans residing in the mainland—to each other and to that of other older US populations to investigate drivers of older age disability.

## Background

### Disability Prevalence among the Older Adult Puerto Rican Population

With the aging of the baby-boomer population in the United States, significant research attention focuses on predictors of their well-being. The share of Latinos in this birth cohort is growing rapidly; in 2012, Latinos made up just 7.3% of those age 65 and older in the United States, but by 2050, an estimated 15 million Latinos will comprise 18% of this demographic (Ortman et al., 2014). The expanding literature on the older Latino population in the United States reflects this demographic reality; heterogeneity by nativity and Latino subgroup is beginning to be explored more fully (C. García, Garcia, & Ailshire, 2018). However, although Puerto Ricans on the archipelago are the third largest group of Latinos in the United States (after Mexicans and Puerto Ricans on the mainland), they are largely absent from research on older Latino health in the United States. There is also limited research comparing the health of older Puerto Ricans residing on the mainland and on the archipelago. Addressing the gap in literature on older Puerto Rican health is particularly important considering the fact that Puerto Ricans may have distinctive health trajectories due to their unique sociopolitical history, the colonial status of the archipelago, and the fact that they are a population experiencing rapid aging partially due to outmigration of working-age individuals (Matos-Moreno, Verdery, et al., 2022). Understanding the health and aging experience of the Puerto Rican population also has important implications for government programs (e.g., Medicare, Social Security) and family-based care, including long-term care arrangements.

Existing research provides evidence of adverse health among older Puerto Ricans. On the mainland older Puerto Ricans have a high prevalence of asthma, cancer, cardiovascular disease, diabetes, HIV, and hypertension (Burgos et al., 2017), morbidity life expectancy (M. A. Garcia et al., 2018) and chronic disease (C. Garcia, Garcia, & Ailshire, 2018; Zsembik & Fennell, 2005). Research on the Puerto Rican archipelago-residing population is even more limited, but documents high rates of hypertension, diabetes, heart disease, and obesity among adults 65 and older on the archipelago (García et al., 2021; Pérez & Ailshire, 2017) and identifies diabetes as a correlate of disability for the archipelago-residing population with an additive effect of comorbid diabetes and depression on the likelihood of disability (Downer et al., 2017). These indicators of high rates of morbidity in the Puerto Rican older adult population both on the US mainland and the archipelago have important implications for disability, defined as difficulty undertaking tasks or roles independently due to health (Verbrugge, 2016). Conceptualized as the “morbidity process” (Crimmins et al., 2010), population-level age-trajectories of health change begin with physiological dysregulation, then diagnosis of diseases and conditions, and subsequently disability and functional loss. Death is the end stage of the “morbidity process.” Therefore, high rates of chronic disease are an upstream indicator of high rates of disability. This has been substantiated empirically in the Puerto Rican population on the mainland where a history of chronic disease including stroke, arthritis, diabetes, depression, heart attack, high BMI, cataract, or respiratory disease is associated with high disability rates (Castaneda-Sceppa et al., 2010; Tucker et al., 2000).

There is also evidence of a disadvantage among Puerto Rican older adults in terms of disability. Using US Census Bureau measurements of disability Markides et al. (2007) find that older Puerto Ricans on the mainland have the highest disability rates among Latino subgroups. Using similar measures but updated data from the Census Bureau, Sheftel (2017) looks at disability prevalence by Latino country of origin and nativity for both the working-age population (18–64) and the older population (65 and older) in the United States. She finds that irrespective of place of birth (archipelago or mainland), Puerto Ricans residing on the mainland have the highest age-standardized disability rates. This Puerto Rican disadvantage compared to other US-born and foreign-born Latinos persists into older ages, with the highest age-standardized disability rates among mainland- and archipelago-born Puerto Rican women. A disability disadvantage among Puerto Ricans residing on the mainland is also found when using the National Health Interview Survey (NHIS) to look at functional limitations, activities of daily living (ADL) limitations, and instrumental activities of daily living (IADL) limitations (Coustasse et al., 2009; Hajat et al., 2000; Melvin et al., 2014). The only exception to this are findings by Mehta et al. (2013) also using NHIS data who find that ADL and functional limitations do not differ between Puerto Ricans and US-born Mexicans.

To our knowledge, only three papers document older adult disability among Puerto Ricans on the archipelago. Payne (2018) compares disability-free life expectancy (DFLE) at age 65 in Puerto Rico to the United States, Mexico, and Costa Rica using data from the Health and Retirement Study (HRS) sister studies.^2^ Payne estimates that 14% of archipelago-residing Puerto Ricans 65 and older report at least one activity of daily living disability and at age 65 women (men) in Puerto Rico can expect to live 20 (18) more years, with about 15 (14) of those years disability-free. Payne finds little variation across countries in the proportion of remaining life spent disabled, despite differences in cross-national economic, public health, nutritional and epidemiological profiles. Prina et al. (2020) estimate consistent DFLE at age 65: 14.1 years for archipelago-residing Puerto Rican men and 16.2 years for women. Additionally, they estimate 14.6 years of dependency-free life expectancy (DepLE) at age 65 for archipelago-residing Puerto Rican men and 17.1 for women. Pérez and Ailshire (2017) find that archipelago-residing Puerto Ricans are less likely to report an activity limitation compared to US-born, mainland-residing non-Hispanic whites.

Taken together, previous research indicates that Puerto Ricans residing on the mainland are particularly vulnerable to older age disability and that archipelago-residing Puerto Ricans have comparable disability rates to the older US population as well as to other Latin American and Caribbean older adult populations. More research, using large-scale, nationally representative data from both the mainland and archipelago, is needed to comprehensively compare disability prevalence within the Puerto Rican population by place of residence and place of birth to understand health determinants at older ages.

### Sources of Variation in Puerto Rican Older Adult Disability

The sociomedical model of disability, or the Disablement Process (Verbrugge & Jette, 1994), provides a useful conceptual scheme to understand potential sources of variation for Puerto Rican older adults by place of birth and place of residence. This model highlights the centrality of risk factors in the pathway from pathology to disability, or the “morbidity process.” That is, certain demographic, social, lifestyle, behavioral, psychological, environmental, and biological characteristics can either increase or decrease the probability that biochemical or physiological abnormality (i.e. pathology) will lead, not only to impairment and functional limitation, but to disability (Verbrugge & Jette, 1994). Here, we focus on three domains that may vary by place of birth and place of residence for Puerto Ricans, differentially exposing them to risk of disability at older ages: social determinants, life course exposures, and health selection.

The following section summarizes the theoretical and empirical evidence related to the three domains of risk vis-à-vis Puerto Rican older adult disability by place of birth and place of residence. This summary is meant to inform our expectations about the *overall* comparative disability prevalence for the three Puerto Rican focal populations analyzed in this paper: archipelago-born/residing (“stayers in PR”), mainland-born/residing (“stayers in US”) and archipelago-born/mainland-residing (“movers”) Puerto Ricans. This review does not purport to be comprehensive of *all* potential sources of variation in risk, nor detail all causal pathways, but instead reflects central drivers of variation that have been identified conceptually and backed by empirical evidence. Table 1 provides a synthesis of the knowledge reviewed in this section. Using plus, minus, and equal signs, the table lists how prevalent each risk factor is expected to be among “stayers in PR” and among “movers” relative to the reference group “stayers in US,” net of all other factors. For example, a plus sign for one group under socio-economic status (SES) means that that group is expected to have a higher average SES than the reference group.

**Table 1.** Factors Contributing to Health Outcomes by Place of Birth/Residence for the Puerto Rican Population.

Factor \ population	Archipelago-born and residing (*Stayers in PR*)	Mainland-born and residing (*Stayers in US)*	Archipelago-born and mainland-residing (*Movers*)
I. Social determinants		Reference population	
Socio-economic status (SES)	–		–
Educational attainment	–		?
Exposure to stressors			
Colonialism	–		–
Discrimination	+		=
Acculturation	+		–
Health			
Access/quality care	–		=
Healthy behaviors	++		+
II. Life course exposure			
Childhood/early life	–		–
Adult/late life	–		=
III. Selection			
Early life mortality disadvantage	+		+
Migration selection			
Healthy migrant	?		?
Era of migration	n/a		Before 1950 + 1950–1980 – 1980–2017 ?
Age at migration	n/a		Child/youth =

*Notes:* Stayers in the mainland US are the reference group. Plus (+) denotes that the factor is expected to be more present, on average, in a given group relative to the reference group, net of all other factors. Likewise, minus (−) denotes that a factor is expected to be less present and equal (=) denotes no comparative difference. Question mark (?) indicates inconclusive evidence. Multiple symbols indicate a much greater (++) or much lesser presence (--).

**Social determinants.** There are three broad categories of social determinants of older adult health: socio-economic status, exposure to stressors, and health access and behaviors (Boen & Hummer, 2019). Socio-economic status and exposure to discrimination and colonialism, both key social determinants of health, are considered “fundamental causes” of health because their association with health persists over time and across populations even though intermediate mechanisms may differ (Link & Phelan, 1995; Phelan & Link, 2015). Exposure to the ongoing stress of disadvantage and discrimination across the life course may have a “weathering” effect (Geronimus et al., 2006) leading to a cumulative disadvantage: heightened disability and wider disparities compared to other groups at older ages (Crystal et al., 2017; Dannefer, 2003; O’Rand, 1996). We expect exposure to these factors and their impact on disability to differ for Puerto Ricans not only by place of birth/place of residence but also by gender. Taking an intersectional approach (Crenshaw, 1991), nativity, ethnicity, and gender intersect to jointly structure health (Brown, 2018; Mullings & Schulz, 2006; Warner & Brown, 2011) and there is empirical evidence that Latino women, especially those born outside the mainland US, experience a double disadvantage in terms of health (Erving & Frazier, 2021).

*Socio-economic status (SES)* is recognized as a predominant factor in subpopulation health variation (Link & Phelan, 1995; Marmot & Wilkinson, 1999) and is a key driver of older adult health disparities by race/ethnicity in the United States (Boen & Hummer, 2019). Resources associated with higher SES enable individuals to avoid risk and access health care and other health-protective factors. For example, more socio-economic resources enable better access to health care which, in turn, prevents or decreases the impact of health conditions leading to less physiological dysregulation and disease onset, and then lower disability at older ages (McMaughan et al., 2020). Educational attainment is another important socio-economic resource that drives pathways to older adult disability (Townsend & Mehta, 2021). Educational attainment impacts the type of occupation that an individual holds over their life course, with employment in occupations involving manual labor more likely to expose individuals to physical risk and result in disability. Likewise, higher educational attainment is associated with higher income and with knowledge of health-related behaviors. Both—higher income and greater health-related knowledge—are associated with a healthier lifestyle (healthy diets, leisure time to exercise, less stress) and decreased prevalence of obesity and smoking (which drive physiological dysregulation and disease onset), and subsequently lower disability at older ages (Townsend & Mehta, 2021). While not exhaustive, these are some of the pathways through which variation in SES by place of birth and place of residence may impact Puerto Rican disability at older ages.

SES disparities characterize Puerto Ricans, both mainland- and archipelago-born and residing. On the mainland, Puerto Ricans have higher rates of poverty, homeownership (Noe-Bustamante et al., 2019), and residential segregation than other US Latinos (Santiago & Galster, 1995; Wahl et al., 2007). Burgos and Rivera (2012) find that residential segregation among Puerto Ricans on the mainland is a powerful structural determinant of disability as it increases the probability of disability, is associated with lower SES, and impacts disability directly and indirectly through SES. Even so, the archipelago is characterized by higher rates of poverty and unemployment than the mainland (Benson, 2020; Kaiser Family Foundation, 2017; Portela & Sommers, 2015), some of which has been driven by limited resource allocation for preventative health and divestment from social assets, including health insurance, due to austerity programs legislated by the United States (without Puerto Rican representation). As such, forces of colonialism have led to reduced access to health care for both disease prevention and intervention on the archipelago (Ramos et al., 2022). Comparing educational attainment, an important indicator of SES, of Puerto Ricans by place of residence, mainland-residing Puerto Ricans are more likely to have at least a high school degree than those residing on the archipelago^3^ (Collazo et al., 2010; Lopez & Velasco, 2011). This means that stayers in Puerto Rico are likely to have lower educational attainment than stayers in the US. The relative educational attainment of movers is less clear and depends on their age at relocation and where they were educated (a factor we address in terms of migration selection). Overall, this evidence points to a disadvantage in terms of SES for archipelago-born Puerto Ricans compared to mainland-born Puerto Ricans, with those residing on the archipelago into older ages at the greatest disadvantage.

These socio-economic factors are not divorced from exposure to *stress*, another social determinant of health (Braveman et al., 2011). Exposure to chronic stress has adverse implications for the neuroendocrine, inflammatory, immune, and vascular systems. This physiological dysregulation may lead to disease and disability. In addition to the possibility of stress from socio-economic deprivation, depending on place of birth and place of residence, Puerto Ricans may be exposed to stress from three sources over their life course. First, Puerto Rico is an unincorporated territory of the United States; residents of the archipelago, while natural-born citizens of the United States, do not have rights equal to those of US citizens residing on the mainland and the Puerto Rican government does not have autonomy over government affairs. Colonialism has long been documented as contributing to health disparities (Axelsson et al., 2016; Czyzewski, 2011; Turshen, 1977). The colonial status of the archipelago is the source of considerable environmental stress in terms of quality of life, socio-economic mobility, and health equity (Baker, 2002; Benach et al., 2019; Duany, 2002). We expect that both stayers in Puerto Rico and movers will have experienced more stress due to the colonial status of Puerto Rico, with those residing on the archipelago into older ages exposed to the most stress.

Second, on the mainland, the long history of discrimination of Puerto Ricans (Burgos et al., 2017) means they are exposed to stress associated with discrimination and racism. Discrimination is a social stressor that induces a physiological response that can be detrimental to health (Clark et al., 1999). In addition to heightened stress, which is a pathway in and of itself to adverse health conditions and disability, discrimination and racism are associated with negative physical and mental health outcomes on the institutional/population level by structuring access to resources associated with better health and on the individual level by denying care or providing worse care based attributed characteristics (e.g., race, ethnicity) (Krieger, 2014; Pascoe & Smart Richman, 2009; Williams et al., 1997). We expect Puerto Ricans residing on the mainland, irrespective of place of birth, to be exposed to this type of stress. Third, archipelago-born Puerto Ricans who move to the mainland may be exposed to migration-related stress. Although Puerto Ricans moving to the mainland do not face the same political incorporation processes as immigrants to the United States, in relocation they do cross geopolitical, social, racial, ethnic, and cultural borders (Aranda, 2007; Duany, 2002), a process which is associated with stress and adverse health (Alcántara et al., 2014), in many cases through perceived discrimination (Torres et al., 2012). Not only do we expect movers to be exposed to stress from the migration process, there is evidence that stayers in the United States may also experience acculturative stress (Capielo et al., 2015; Mena et al., 1987) despite not having moved to the mainland themselves.

*Access to and quality of care* and *health behaviors* operate both in conjunction with SES and discrimination, and independently, as social determinants of health. Residents of the archipelago are more likely to be insured, to report a usual source of care, and to have had a check-up in the last year than mainland residents, but this is partially explained by more readily available Medicaid coverage (Portela & Sommers, 2015). Despite advantages in insurance coverage, the quality of care is generally lower on the archipelago (Rivera-Hernandez et al., 2016) and preventative care is poor (Payne, 2018). Significant challenges to the Puerto Rican healthcare system include inadequate funding, outmigration of physicians (Pérez & Ailshire, 2017), lack of low-income subsidies for prescription drugs which leads patients to forgo medications (Perreira et al., 2017), and uneven distribution of healthcare facilities, especially in rural areas (García et al., 2021). Rivera-Hernandez et al. (2016) compare Medicare Advantage enrollees in the United States and Puerto Rico and find that enrollees in PR received worse care on the majority of performance-based quality measures related to chronic disease. Overall, this points to a greater risk of inadequate quality of care for stayers in PR, compared to both groups of mainland-residing Puerto Ricans, despite evidence of higher rates of insurance coverage. Notwithstanding these disparities in healthcare quality on the archipelago, Puerto Rico may be a healthier context for aging than the mainland due to lower rates of poor health behaviors including smoking and obesity (Payne, 2018) and an emphasis on kin-based caregiving, or familism, which provides support and resources to maintain good health (Pérez & Ailshire, 2017). This suggests that those born on the archipelago (stayers in PR and movers) may have more protective health behaviors than stayers in the US.

**Life course exposure.** Exposure to socio-economic factors, sources of stress, and healthcare access, quality, and behaviors will differ for Puerto Ricans by place of birth and place of residence because older adult health outcomes are driven by both *childhood and adult exposure to social determinants* (Payne, 2018). Life course epidemiology emphasizes the central role of childhood exposures on older adult health (Kuh et al., 2003) with evidence of a “long arm” of childhood health and SES on older adult functional limitation (Haas, 2008). Those born on the archipelago, particularly rural archipelago residents, were likely exposed to more adverse early life conditions than mainland-born Puerto Ricans. Prenatal exposure to poor nutrition and infectious disease among archipelago-born Puerto Ricans who resided in the countryside for a prolonged period during childhood is associated with a higher prevalence of older adult heart disease (McEniry et al., 2008; McEniry & Palloni, 2010). Poor childhood conditions of archipelago-born Puerto Ricans are also associated with a higher probability of disability at older ages, with chronic disease representing the main pathway through which adverse early conditions impact physical functioning (Monteverde et al., 2009). Beyond early life conditions, access to advanced medical care and health behaviors during adulthood also impact older adult health outcomes (Payne, 2018). Archipelago-born and residing older individuals have less access to quality health care and advanced medical technology resulting in a higher likelihood of disability (Cutler, 2001).

**Health selection.** Selection plays a role in differential older age disability of Puerto Ricans by place of birth and place of residence. First, *mortality selection* may operate differently for archipelago- and mainland-born Puerto Ricans. Despite recent improvements in infant and childhood mortality rates in Puerto Rico, rates are still higher than on the mainland (Kaiser Family Foundation, 2017). Older individuals born on the archipelago represent cohorts who survived high infant and child mortality rates (Payne, 2018) and thus both movers and stayers in PR may be more positively selected on health than stayers in the US (Gerst-Emerson et al., 2015).

Second, *health-selective migration* may drive differences in older adult disability risk. Because archipelago-born Puerto Ricans can relocate to the mainland without restriction, the direction of bias is unclear. On the one hand, it may be the case that Puerto Ricans relocating from the archipelago to the mainland do so to access better medical care and thus are negatively selected on health (Zsembik & Fennell, 2005), a situation similar to the “sick immigrant effect” found among Jewish immigrants to Israel (Constant et al., 2018). This is the opposite of the positive health selection that operates for Latino immigrants to the United States (C. García, Garcia, & Ailshire, 2018). On the other hand, there is some evidence, based on infant mortality rates, that Puerto Ricans who move from the archipelago to the mainland are positively selected on health compared to non-movers (Landale et al., 2000, 2006).

In terms of *SES-selective migration*, the composition of movers may differ based on era of first migration and age at first migration. Previous research indicates that before 1950, those moving to the mainland were primarily educated and skilled and could afford the relatively high transportation costs. The composition of Puerto Ricans moving from the archipelago to the mainland changed around 1950 as a result of Operation Bootstrap. Implemented in 1947, Operation Bootstrap was an industrialization program that aimed to transform Puerto Rico’s agrarian-based economy and resulted in mostly urban-centered industry, internal migration from the countryside to urban areas, and high rates of unemployment into the 1960s (Dietz, 1986). Combined with reductions in the cost of travel, movers from the 1950s through the 1980s were largely unskilled and low-income, seeking employment opportunities on the mainland. Since 1980 the flow of movers from the archipelago to the mainland has been more representative of all Puerto Rican social classes (Aranda, 2007; Grosfoguel, 1999). Additionally, evidence from other Latino populations indicates that age at migration is an important determinant of older adult disability (M. A. Garcia et al., 2017; M. A. Garcia & Chiu, 2016; M. A. Garcia & Reyes, 2018). While the application to the Puerto Rican population, which is more transitory, is unclear, archipelago-born Puerto Ricans relocating to the mainland as children are more likely exposed to similar risk factors as stayers in the US, compared to movers relocating at older ages.

This cumulative review of sources of variation impacting Puerto Rican health frames our largely descriptive comparative analysis of disability by place of birth and place of residence. As evidenced by Table 1, both groups of archipelago-born Puerto Ricans have more risk factors for disability at older ages compared to mainland-born Puerto Ricans. Informed by the weathering framework (Geronimus et al., 2006), we thus expect archipelago-born Puerto Ricans to experience higher rates of disability at older ages. This is consistent with previous research finding higher disability prevalence among archipelago-born Puerto Ricans residing in the United States compared to mainland-born and residing Puerto Ricans (Heron et al., 2003; Sheftel, 2017). Due to data limitations, we are unable to parse out the exact contribution of each factor, nor account for mechanisms overlooked in the existing literature. Instead, our analysis does the following: first, we determine if the overall prevalence of disability by group conforms to our hypotheses of comparative risk. Second, we look specifically at two potential drivers—educational attainment and era of migration.

## Data and Methods

### Data

We use publicly accessible data from the American Community Survey (ACS) and Puerto Rico Community Survey (PRCS) 2013–2017 5-year samples^4^ (Ruggles et al., 2019) retrieved from the IPUMS Web site. Annually, the ACS randomly samples one percent of the population of the 50 US states, and the PRCS, customized for Puerto Rico, annually takes a random sample of 36,000 housing unit addresses of the archipelago-based Puerto Rican population. They are both offered in English and Spanish.

We restricted the analysis to specific groups based on race, ethnicity, and nativity and use categories and terminology consistent with the ACS and PRCS surveys. The surveys ask about Hispanic origin, race, and place of birth. Respondents of Hispanic, Latino, or Spanish origin are prompted to provide further detail and indicate if they are of Mexican, Puerto Rican, Cuban, or other Hispanic origin. The focus of this paper is on three^5^ populations: mainland-born Puerto Ricans residing in the mainland, archipelago-born Puerto Ricans residing on the archipelago, and archipelago-born Puerto Ricans residing in the mainland. For simplicity of terminology, we refer to each of these three groups respectively as stayers in the US, stayers in PR, and movers*. Stayers in the US* are those who indicate that they were born in one of the 50 US states, indicate they are Latino of Puerto Rican origin, and were sampled by ACS 2013–2017. *Stayers in PR* are those who indicate that they were born in Puerto Rico, indicate that they are Latino of Puerto Rican origin, and were sampled by PRCS 2013–2017. *Movers* are those who were born in Puerto Rico, indicate that they are Latino of Puerto Rican origin, and were sampled by ACS 2013–2017.

Additionally, we include several comparison populations all drawn from the ACS sample. US-born non-Hispanic Whites are those who report their race is White, that they are not of Hispanic origin, and were born in the 50 US states. US-born non-Hispanic Blacks are those who report that their race is Black, that they are not of Hispanic origin, and were born in the 50 US states. US-born of Mexican origin are those who report that they are Latino of Mexican origin and were born in the 50 US states, and foreign-born Mexicans are those who report that they are Latino of Mexican origin and were born in Mexico. Further, we restrict the sample to those age 40 and above.

### Measures of Disability

Census Bureau disability measures (e.g., ACS) are generally considered reliable for studying US disability patterns and trends (Elo et al., 2011; Erikson, 2012; Gubernskaya et al., 2013; Markides et al., 2007; Siordia, 2016; Siordia & Ramos, 2015). The survey includes six disability questions regarding hearing, vision, cognitive, ambulatory, self-care, and independent living difficulties, covering a wide range of activities and health-related difficulties. We construct a binary measure of *overall disability* based on individuals’ responses to the six disability questions on the ACS survey (coded 1 if the individual answered affirmative to at least one of these six questions).

In addition to *overall disability prevalence rates (“any disability”)*, we conduct separate analyses of the six underlying disability domains. We also analyze *total disability* rates, calculated using the total number of disabilities reported across the six domains. Results from these supplementary analyses did not differ meaningfully from patterns of overall disability. Results are available upon request.

#### Analytic Strategy

To estimate age-specific rates of disability for various groups using graphical representation with 95% Confidence Intervals, the individual-level data were aggregated by 5-year age groups, using ACS-provided person weights, *perwt,* normalized using Stata’s *analytic weight* function. The analysis of prevalence patterns conducted using graphs of age-specific disability rates is complemented by regressions using gender-specific linear probability models of whether or not a person had a disability. Informed by an intersectionality approach (Crenshaw, 1991) which holds that health is jointly structured by the intersection of nativity, ethnicity, and gender (Brown, 2018; Mullings & Schulz, 2006; Warner & Brown, 2011), and due to empirical evidence of gender-specific aging trajectories and higher disability prevalence for Puerto Rican women compared to men (Melvin et al., 2014; Sheftel, 2017), all analyses are stratified by gender.

Focusing on age-specific prevalence allows us to study disability patterns adjusted for differences in age structure across the underlying populations. Such differences do exist (see next section) and they have been shown to meaningfully affect aggregate disability patterns based on crude rates (Sheftel, 2017; Sheftel & Heiland, 2018).

To investigate the contribution of educational attainment and era of migration, we estimate age-specific disability prevalence by education level and migration era. Education is dichotomized into those with less than a high school degree and those with a high school degree, GED or more. We also investigated era of first migration to the mainland for movers, creating a categorical variable informed by important differences in migration stream selectivity detailed previously: arriving before 1950, between 1950 and 1979, and arriving in 1980 or later.

## Findings

### Age-specific Disability Prevalence: in Comparison

The focus of this analysis is on three groups of Puerto Rican origin and includes four comparison groups. Table 2 presents summary statistics of key variables, separately for women and men, for these seven groups as well as the overall analytic sample. Males represent 47.6% of the overall analytic sample, with the lowest concentration of males among the archipelago-born populations. The archipelago-born Puerto Rican groups and US-born Whites have the oldest average ages of 60–62, while mainland-born Puerto Ricans are the youngest at 52–53 years old. Of the Puerto Rican groups those born on the archipelago have a considerably higher concentration of individuals without a high school degree (32–34% for movers and 29–33% for stayers in PR) as compared to those born on the mainland (14–18%).

**Table 2.** Sample Descriptives (Weighted), Women.

	Female Freq	Age	% edu < HS	% disability (all)	% disability (age<55)	% disability (55–69)	% disability (>69)
(a) Women							
Race/Ethn./Origin/Location							
US-born NH-White	2,986,129	60.3	7.4	21.1	11.8	18.5	39.9
US-born NH-Black	377,155	57.7	15.0	27.6	17.0	30.0	49.7
US-born Mexican origin	110,480	55.6	22.0	21.1	12.2	24.3	47.5
Foreign-born Mexican	127,481	53.6	61.3	15.0	6.9	19.6	48.6
US mainland-born Puerto Rican (“stayer in US”)	19,153	52.1	13.8	21.9	17.4	27.0	49.4
Archipelago-born Puerto Rican in US mainland (“mover”)	21,720	60.0	33.2	33.7	20.6	34.1	53.1
Archipelago-born Puerto Rican in Puerto Rico (“stayer in PR”)	40,330	60.6	28.6	34.9	20.2	33.3	57.2
Sum/average	3,682,448	59.4	11.7	21.9	12.3	20.4	41.6
(b) Men							
Race/Ethn./Origin/Location							
US-Born NH-White	2,745,022	59.1	8.5	21.4	11.8	21.0	40.4
US-Born NH-Black	304,542	56.4	18.1	26.0	17.5	30.3	43.9
US-Born Mexican origin	99,413	54.6	22.4	21.9	13.2	27.4	48.7
Foreign-born Mexican	126,119	52.6	61.7	12.3	6.1	17.5	44.4
US Mainland-born Puerto Rican (“stayer in US”)	17,257	51.6	17.3	19.9	16.2	25.8	39.2
Archipelago-born Puerto Rican in US mainland (“mover")	18,146	58.7	34.5	28.6	19.3	31.0	43.0
Archipelago-born Puerto Rican in Puerto Rico (“stayer in PR")	32,139	59.7	33.2	33.8	21.6	35.5	51.1
Sum/Average	3,342,638	58.3	13.3	21.6	12.3	22.3	41.1

Table 2 also presents disability prevalence rates overall and by age (age <55, 55–69, and 70+) for all groups included in the analysis. As expected, disability increases across age categories for all groups, especially between ages 55–69 and 70+. Regardless of gender, mainland-born Puerto Ricans are significantly less likely to have any disability than their archipelago-born counterparts. Among mainland-residing Puerto Ricans (movers and stayers in the US) females tend to have higher overall disability rates than males at ages 55–69 and past age 70.

**Figure 1.**
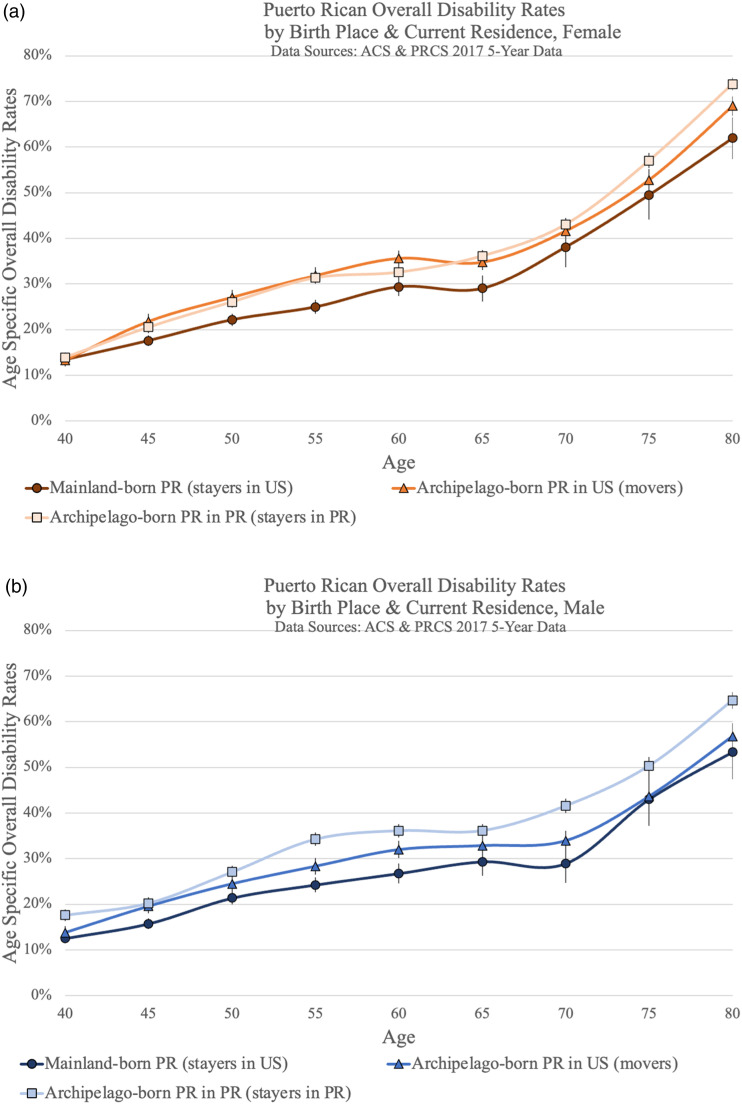
Puerto Rican overall disability rates by birth place and current residence, (a) female, (b) male.

Figures 1(a) and 1(b) visualize overall disability rates for all three Puerto Rican groups by gender respectively. For both females and males, stayers in the US have the lowest overall disability rates across the age spectrum. For women, there is no statistically significant difference between movers and stayers in Puerto Rico. Male stayers in PR have the highest disability rates at ages 55–64 and 70–74.

**Figure 2.**
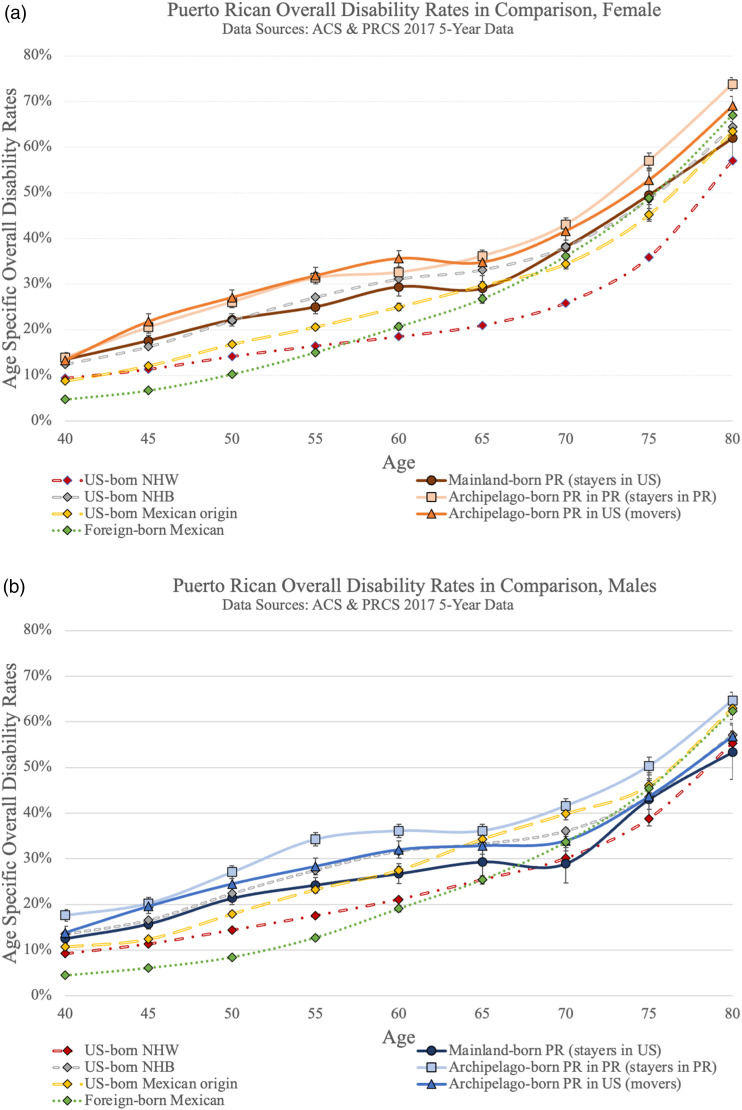
Puerto Rican overall disability rates in comparison (a) female, (b) male.

Figures 2(a) and 2(b) add disability prevalence rates for comparison populations by gender. Among females, as visualized in Figure 2(a), archipelago-born Puerto Ricans (movers and stayers in PR) have the highest overall disability rates of all the populations until age 60 when confidence intervals overlap with US-born non-Hispanic Black females. Mainland-born Puerto Rican females also have comparatively high overall disability rates, comparable to US-born non-Hispanic Blacks and higher than all the other non-Puerto Rican populations. For males, visualized in Figure 2(b), similar patterns hold but statistically significant differences between groups are not as prevalent. Until age 54, all groups of Puerto Rican origin as well as Blacks have the highest disability rates.

### Disability Prevalence by Education and Era of Migration

**Figure 3.**
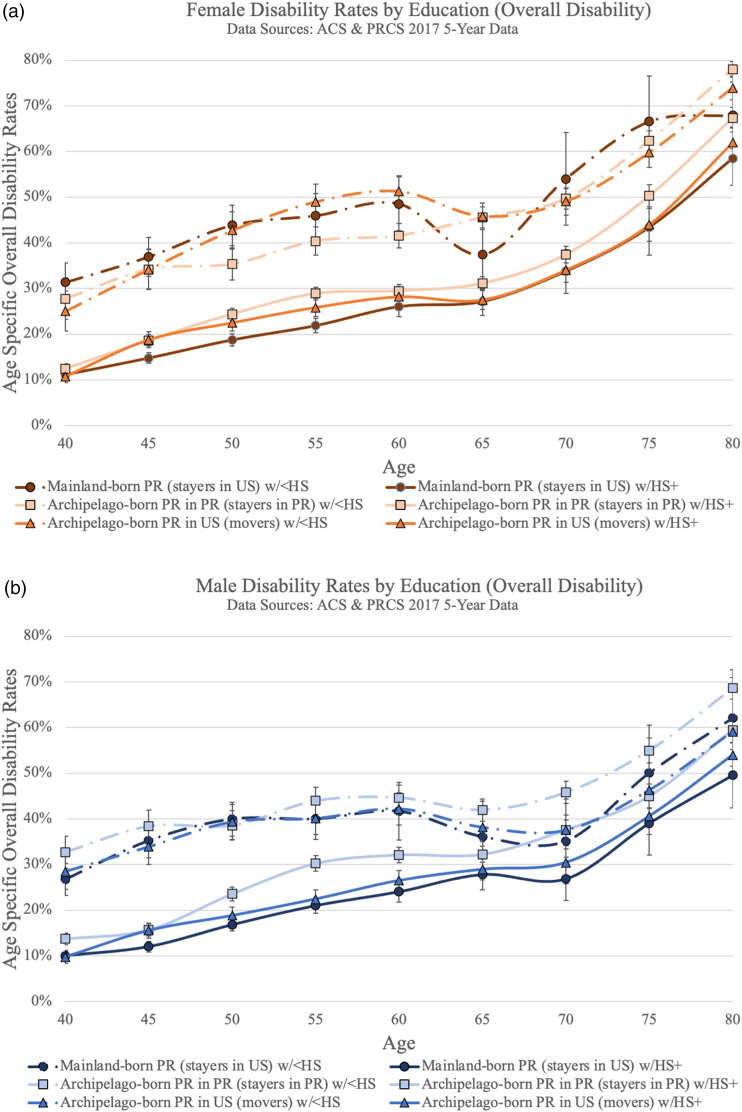
(a) Female disability rates by education (overall disability), (b) male disability rates by education (overall disability).

In trying to understand underlying processes leading to these results, we estimated disability prevalence rates for the Puerto Rican origin groups both by level of education and era of migration. Figures 3(a) and 3(b) visualize disability prevalence rates by education level, separating those who have a high school degree or higher from those who do not. As expected, for both females and males more education is associated with lower disability prevalence rates. For females (Figure 3(a)) this is true across the age spectrum. By age 65, male stayers in Puerto Rico (Figure 3(b)) with a high school degree or more have overlapping confidence intervals with stayers in the US with less than a high school degree.

Within educational attainment groups the relative comparison between movers and both groups of stayers changes. Because sample sizes are relatively small, confidence intervals often overlap, especially at older ages; however, some important patterns are critical to note. For those with higher educational attainment, both male and female stayers in the US remain the least disabled. However, among those without a high school degree, both male and female stayers in the US do not exhibit a clear disability advantage as they do before disaggregation by education.

Regression results confirm the patterns found in the age-specific graphical analysis. Table 3 shows estimated coefficients from gender-specific linear probability regressions of overall disability on race/ethnicity/origin/location with and without controls for age and educational attainment (less than high school degree). Results from Models 1 and 2 show that age differences explain part of the overall gap in disability prevalence among Puerto Ricans. Stayers in Puerto Rico tend to be younger than both movers or stayers in the US (see Tables 2a and 2b), and they experience lower disability prevalence on average partly because of that age advantage. Model 3, which adds a control for lack of a high school degree to Model 2, shows that differences in education explain part of disability disparity between the mainland- and archipelago-born Puerto Ricans that remained in Model 2. Specifically, the results in Model 2 suggest that, adjusted for age, females (males) stayers in the US are 5.7 (5.2) percentage points less likely to have a disability than stayers in Puerto Rico. Additionally controlling for the higher educational attainment of the mainland-born population in Models 3 (see Tables 2), this gap is reduced to 3.3 (2.9) percentage points for females (males).

**Table 3.** Estimates From ACS-PRCS Descriptive Regressions Weighted, Any Disability.

	Women			Men		
	Model 1	Model 2	Model 3	Model 1	Model 2	Model 3
Race/Ethnic/Origin/Location						
Ref.: US mainland-born Puerto Ricans						
US-Born NH-White	−.008**	−.077***	−.060***	.015***	−.051***	−.033***
	(.004)	(.004)	(.004)	(.004)	(.004)	(.004)
US-Born NH-Black	.056***	.012***	.013***	061***	.021***	.021***
	(.004)	(.004)	(.004)	(.004)	(.004)	(.004)
US-Born Mexican origin	−.008**	−.038***	−.051***	.021***	−.007*	−.014***
	(.004)	(.004)	(.004)	(.004)	(.004)	(.004)
Foreign-born Mexican	−.070***	−.168***	−.168***	−.076***	−.086***	−.159***
	(.004)	(.004)	(.004)	(.004)	(.004)	(.004)
Island-born Puerto Rican in US mainland	.117***	.053***	.022***	.087***	.026***	.000
	(.006)	(.005)	(.005)	(.006)	(.006)	(.006)
Island-born Puerto Rican in Puerto Rico	.129***	.057***	.035***	.139***	.067***	.044***
	(.005)	(.004)	(.004)	(.005)	(.005)	(.005)
*Observations*	3,682,448	3,682,448	3,682,448	3,342,638	3,342,638	3,342,638
*R-squared adj*	.006	.103	0.120	.005	.084	.100
Controls						
Age Dummies 5-yr groups	No	Yes	Yes	No	Yes	Yes
Education < HS	No	No	Yes	No	No	Yes

*Notes*: Robust Standard Errors in parentheses. All models also include a constant term. ***p < .01, **p < .05, *p < .10

Our analysis of disability prevalence by era of migration showed no statistically significant differences by period of arrival for movers. This is despite the fact that the socio-economic composition of Puerto Rican migration flows to the mainland differ by era of arrival. We conjecture about the reasons for this finding in the discussion. Results and graphs are available upon request.

## Discussion

This paper makes a critical contribution to understanding health patterns among an understudied Latino population. Even though Puerto Ricans as a whole (mainland and archipelago) are the second largest Latino group in the United States, making up about 10% of the total Latino population, we know little about patterns and determinants of their health, especially at older ages and for the archipelago-residing population specifically. This gap in research is particularly concerning because the Puerto Rican population on the archipelago is rapidly aging, partially as a result of high rates of outmigration of younger cohorts. Therefore, disability estimates are critical to understanding what the aging of this population represents in terms of current and anticipated pressures on healthcare systems, costs to public programs (e.g., Medicare, Social Security), and family care demands. Considering the unique sociopolitical history and colonial status of Puerto Ricans—including the fact that Puerto Ricans are US citizens who *do not* face barriers to migration between the archipelago and the mainland but who *are* subject to colonialism and unequal rights—generalizing research on disability from other US-born populations or Latino immigrant groups to the Puerto Rican older adult population may be mistaken. This analysis, combining sources of large-scale, nationally representative data on Puerto Ricans from both the archipelago and the mainland US, is therefore critical for policymakers and public health practitioners preparing for the aging of the Puerto Rican population.

First, we provide updated disability prevalence rates for three groups of Puerto Ricans. Among the race, ethnicity, and nativity groups analyzed here, all three Puerto Rican groups have the highest disability rates, comparable to or higher than non-Hispanic Blacks. Considering the three groups of Puerto Ricans, we find that those who are mainland-born and mainland-residing (“stayers in the US”) have the lowest disability rates, particularly at younger ages. This result is in accordance with our expectation of less weathering (Geronimus et al., 2006) of stayers in the US based on lower exposure to socio-economic adversity, colonialism, and discrimination, outlined in our review of the cumulative forces impacting Puerto Rican health (see Table 1). Through the conceptual framework of the sociomedical model of disability (Verbrugge & Jette, 1994) stayers in the US have less overall exposure to risk factors in the pathway from pathology to disability. Table 4 summarizes these results, mirroring Table 1 in structure and direction of expected disability risk. While the contribution of each force cannot be analyzed using these data and is therefore beyond the scope of this paper, it is evident that the health risks associated with social determinants, exposure to stress, and selection mechanisms result in higher disability prevalence among movers to the mainland and stayers in PR.

**Table 4.** Summary of Findings.

	Puerto Rican groups		
	Archipelago-born and residing *(Stayers in PR)*	Mainland-born and residing *(Stayers in US)*	Archipelago-born and mainland-residing (*Movers*)
Male			
Age	+	Reference population	+
Education (by age)			
< HS	=		=
HS+	=		=
Migration era (by age)			
Before 1950	+		+
1950–1980	+		+
1980+	+		+
Female			
Age			
Education (by age)			
< HS	=		=
HS+	+		=
Migration era (by age)			
Before 1950	+		+
1950–1980	+		+
1980+	+		+

*Note*: Although analyses report age-specific (5-yr) disability prevalence, here we summarize overall patterns which may vary slightly by age.

+ denotes higher disability prevalence compared to the reference group (Stayers in the US),

− denotes lower disability prevalence compared to the reference group (Stayers in the US),

= denotes equivalent disability prevalence compared to the reference group (Stayers in the US).

Overall, similarities in disability prevalence are strongest based on where one was born as opposed to current residence. This points to the contribution of early life exposures to adversity, poverty, and colonialism, which are more substantial on the archipelago, to middle and older adult disability prevalence. This finding extends the life course theory emphasis on childhood as a critical period in health trajectories to a new population: Puerto Ricans. Indeed, we find that exposures during childhood have a “long arm” in their impact on later-life health outcomes regardless of adult exposures (Haas, 2008; Kuh et al., 2003). The finding that at older ages—between 75 and 79 for males and 70 and 74 for females—disability rates for mainland-born Puerto Ricans are statistically equivalent to those of the archipelago-born groups also points to another potential process, supported by the weathering hypothesis, whereby chronic and cumulative exposure to social and economic disadvantage across the life course by Puerto Ricans *on the mainland* leads to physical decline (Forde et al., 2019; Geronimus et al., 2006). Concentration in occupations involving heavy labor over time is one such exposure to disadvantage, that may lead to high rates of disability for mainland-born Puerto Ricans at older ages (Pebley et al., 2021).

In addition, comparing Puerto Rican disability rates to those of other US populations, non-Hispanic Blacks are found to be most similar (Figures 2(a) and 2(b)). Within the Black population in the United States, there is a well-established connection between perceived discrimination and physical and mental health outcomes (Williams & Mohammed, 2009). While less studied among the Puerto Rican population (Araújo & Borrell, 2006), this association has also been found (Lee & Ferraro, 2009; Szalacha et al., 2003). In absence of data on perceived discrimination from the ACS and PRCS we cannot assess this association directly, but it is descriptively supported by our findings.

This paper is subject to several limitations. First, the data are cross-sectional and thus do not consider potential cohort effects and cannot directly assess the contribution of early life factors on later-life outcomes. Limited longitudinal data with insufficient sample sizes of our key groups means that we must rely on a cross-sectional analysis to give us first insights into patterns. This limitation adds support to the call for longitudinal data on Latino health allowing an analysis of heterogeneity by country of origin and nativity (Tienda, 2002). Second, this analysis documents disability patterns before Hurricanes Harvey, Irma, and Maria, and does not incorporate the long-term effects of these hurricanes on health in Puerto Rico since 2017 (Alexander et al., 2019; Keenan & Hauer, 2020; Santos-Lozada et al., 2020).

Third, the fact that Puerto Ricans have US citizenship, and thus freedom to move from the archipelago to the mainland, means that their migration patterns are often temporary and circular. Because of the confines of cross-sectional data, the categories by place of residence delineated in this paper are temporally limited. That is, we estimated disability rates of archipelago-born Puerto Ricans who are *in the mainland US at the time of* the survey as compared to disability rates of archipelago-born Puerto Ricans who are *on the archipelago at the time of* the survey. With such a transnational population these are estimates for one point in time and must be considered as such. This complicates estimates of “stayers” in Puerto Rico because it is likely that a considerable portion resided on the mainland at younger ages and subsequently returned. Therefore, migration factors associated with health, which we hypothesize to only impact movers, may also be present for those stayers in Puerto who have previously resided on the mainland. Our findings that disability prevalence is similar between the two archipelago-born populations are consistent with this potential source of bias. This lends support to the fact that our cross-sectional estimates are accurate at older ages.

Additionally, the cross-sectional design of ACS which lacks a longitudinal tracking of circular migration also may explain why the prevalence of disability did not differ by period of migration despite variation in SES. The ACS only collects year of most recent arrival on the mainland and thus those who engaged in circular migration may be misclassified as part of a more recent migration stream. Further, because we see strong evidence of place of birth contributing to later-life disability, it is important to understand how age at migration to the mainland mitigates this relationship. However, the single “most recent year of arrival” is not sufficient to conduct an analysis of age-at-arrival for such a transitory population. We hypothesize that those movers who arrive on the mainland at earlier ages, and remain on the mainland, will have fewer disabilities when older compared to those who arrived at later ages because of less exposure to adverse conditions on the archipelago. Future research, using longitudinal data, should explore these patterns.

This paper makes critical contributions to understanding health disparities among a large and growing but understudied older US Latino population using some of the only large-scale, nationally representative data sources that can be combined from both the Puerto Rican archipelago and the mainland. The high disability prevalence rates among the Puerto Rican population on both the archipelago and the mainland have considerable implications for government planning, spending, and service provision as governments prepare to meet the needs of this rapidly aging population. These results have public health implications, informing potential avenues to reduce disparities. Our findings also extend existing life course theory understandings of the critical role of early life exposures to older adult health. The similar patterns of disability for both groups of archipelago-born Puerto Ricans provide evidence of the long-term health consequences of childhood conditions (Haas, 2008) for older adult disability. These results also highlight that efforts to reduce childhood poverty, increase educational attainment, and improve access to quality health care on the archipelago at younger ages, has implications for health across the life course. This research provides a renewed call for investing in and improving socio-economic conditions in Puerto Rico (including through political representation and decolonialization), which have lasting repercussions for health outcomes throughout the life course. On the mainland, Puerto Ricans, regardless of place of birth, have among the highest disability rates compared to other race/ethnicity/nativity groups. Efforts to decrease Puerto Rican older adult disability disparities should be aimed at reducing residential segregation, improving educational attainment, and reducing the marginalization and discrimination of the Puerto Rican population on the mainland. More generally, this research highlights the problematics of combining heterogenous populations into aggregated groups when assessing health outcomes, which impede an understanding of underlying processes contributing to differences in health patterns and thus the ability to improve modifiable risk factors.

## Notes

1. Based on authors’ analysis of ACS and PRCS 2013–2017 data.

2. See the University of Southern California, Gateway to Global Aging for more information on the HRS family of longitudinal aging studies.

3. There is evidence that educational attainment of Puerto Ricans on the Archipelago is more bifurcated than on the mainland. That is, a relatively large share of the population on the Archipelago do not have a high school degree and as well as a large share have a BA or more. Educational attainment is more equally distributed among the mainland-residing population (Lopez & Velasco, 2011; Mora et al., 2017).

4. The PRCS data for 2017 reflects the population characteristics prior to Hurricane Maria which devastated the Archipelago in late September 2017 (US Census Bureau, 2018).

5. A fourth population, those born in the mainland US who move to Puerto Rico (sampled by PRCS), are not considered in this paper because they are a relatively small population (*N* = 3649) and do not allow an analysis of age-specific patterns, central to this paper.
